# Family meetings and end-of-life decision-making in Thai critically ill patients

**DOI:** 10.1186/cc11096

**Published:** 2012-03-20

**Authors:** P Chatrkaw, R Champunot, W Riyakul

**Affiliations:** 1 Faculty of Medicine, Chulalongkorn University, Bangkok, Thailand; 2 Buddhachinaraj Hospital, Phitsanulok, Thailand; 3 Chulalongkorn Hospital, Bangkok, Thailand

## Introduction

Limitation of life-sustaining therapy after critical illnesses in Thailand is uncommon. The barriers may be uncertain prognosis, wrong sense of doctor duty, guilty feeling, conflicts on the goals of care and fear of liability. Therefore we set a formal healthcare team meeting followed by a family meeting to find the balance between curative and palliative intention. The objective was to determine the nature and effects of family meetings in the Thai social context.

## Methods

A descriptive, retrospective analysis of charts and preference forms after family meetings in the surgical ICU during 2003 to 2005. Close family members were invited and encouraged to express their ideas and feelings.

## Results

**Figure 1:**
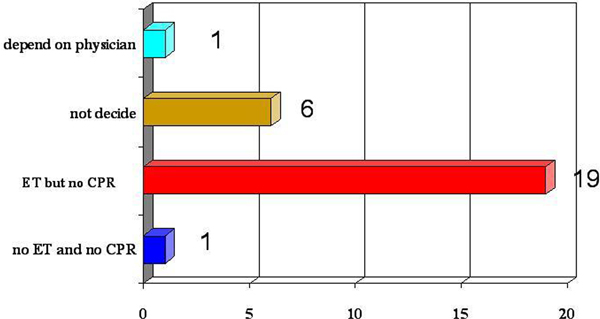
**Preferences for care at the end of life after family meetings**.

Thirty-one family meetings were analysed. The mean age of the patients was 65.5 (± 12.9) with mean SAPS II of 55.6 (± 14.9). Three patients were post CPR. Metastatic cancer was the most common underlying condition (45.2%). Most families requested to have full support, except CPR. Around 20% were not ready to make a decision, but finally agreed not to escalate therapy. See Figure 1.

## Conclusion

Withholding intubation and withdrawal therapy are uncommon in Thai people. However, most Thai families prefer not to escalate therapy including CPR. All of them died peacefully and the families were satisfied with the care at the end of life.
